# Divergence of *Borrelia burgdorferi* sensu lato spirochetes could be driven by the host: diversity of *Borrelia* strains isolated from ticks feeding on a single bird

**DOI:** 10.1186/1756-3305-7-4

**Published:** 2014-01-02

**Authors:** Nataliia Rudenko, Maryna Golovchenko, Natalia M Belfiore, Libor Grubhoffer, James H Oliver Jr

**Affiliations:** 1 Biology Centre AS CR, Institute of Parasitology, České Budějovice, 37005, Czech Republic; 2 James H Oliver, Jr Institute of Coastal Plain Sciences, Georgia Southern University, Statesboro, GA, 30460-8056, USA; 3 Department of Biology, University of Tampa, Tampa, FL, 33606, USA; 4 University of South Bohemia, České Budějovice, 37005, Czech Republic

**Keywords:** *Borrelia burgdorferi* sensu lato, *Ixodes minor*, Bird migration, Bird reservoir host, Multilocus sequence analysis, Multilocus sequence typing, Recombinant genotypes, Southeastern United States

## Abstract

**Background:**

The controversy surrounding the potential impact of birds in spirochete transmission dynamics and their capacity to serve as a reservoir has existed for a long time. The majority of analyzed bird species are able to infect larval ticks with *Borrelia.* Dispersal of infected ticks due to bird migration is a key to the establishment of new foci of Lyme borreliosis. The dynamics of infection in birds supports the mixing of different species, the horizontal exchange of genetic information, and appearance of recombinant genotypes.

**Methods:**

Four *Borrelia burgdorferi* sensu lato strains were cultured from *Ixodes minor* larvae and four strains were isolated from *Ixodes minor* nymphs collected from a single Carolina Wren (*Thryothorus ludovicianus*). A multilocus sequence analysis that included 16S rRNA, a 5S-23S intergenic spacer region, a 16S-23S internal transcribed spacer, *flagellin*, *p66*, and *ospC* separated 8 strains into 3 distinct groups. Additional multilocus sequence typing of 8 housekeeping genes, *clpA, clpX, nifS, pepX, pyrG, recG, rplB,* and *uvrA* was used to resolve the taxonomic status of bird-associated strains.

**Results:**

Results of analysis of 14 genes confirmed that the level of divergence among strains is significantly higher than what would be expected for strains within a single species. The presence of cross-species recombination was revealed: *Borrelia burgdorferi* sensu stricto housekeeping gene *nifS* was incorporated into homologous locus of strain, previously assigned to *B. americana*.

**Conclusions:**

Genetically diverse *Borrelia* strains are often found within the same tick or same vertebrate host, presenting a wide opportunity for genetic exchange. We report the cross-species recombination that led to incorporation of a housekeeping gene from the *B. burgdorferi* sensu stricto strain into a homologous locus of another bird-associated strain. Our results support the hypothesis that recombination maintains a majority of sequence polymorphism within *Borrelia* populations because of the re-assortment of pre-existing sequence variants. Even if our findings of broad genetic diversity among 8 strains cultured from ticks that fed on a single bird could be the exception rather than the rule, they support the theory that the diversity and evolution of LB spirochetes is driven mainly by the host.

## Background

The list of hosts for *Ixodid* ticks that serve as reservoirs for *Borrelia* currently includes several hundred vertebrate species comprised of mammals, reptiles and birds [1]. The controversy surrounding the ability of birds to serve as reservoirs, and the impact of birds in spirochete transmission dynamics has existed for quite a long time [2-5]. Recent findings indicate that the majority of analyzed bird species are able to infect larval ticks with *Borrelia*[1,6-8]. However, reservoir capabilities of different bird species vary, as they do in mammals [9]. Infection rates in ticks removed from birds is comparable to those removed from mammals and in some cases reaches as much as 43.5% [9,10]. At least 80 bird species parasitized by Ixodid ticks are recognized in North America [6,11]. Additionally, 300 seabird species are involved in a global transmission cycle [12]. The role of birds in the spread of infected ticks is now well documented [12-14]. Earlier estimations have revealed, for example, that birds disperse 50 to 175 million *Ixodes scapularis* ticks across Canada each spring [15]. Today, the reservoir role of various bird species, especially those of ground-nesting and ground-foraging birds, such as thrushes, blackbirds, robins, wrens, nightingales, blue throats and pheasants, is unanimously recognized. They transport infected ticks and the pathogens, and thus serve as efficient amplifying reservoirs of some spirochete species worldwide [10,15-17].

Nineteen named spirochete species from the *Borrelia burgdorferi* sensu lato (sl) complex are recognized around the world [18-34]. The development and wide usage of new techniques such as multi locus sequence analysis (MLSA) [28] and multi locus sequence typing (MLST) [35] resulted in the description of 6 new spirochete species in the past 5 years, *B. yangtze*[20], *B. carolinensis*[31], *B. bavariensis*[26], *B. americana*[32], *B. kurtenbachii*[25] and *B. finlandensis*[34]. Divergences among known *Borrelia* species reflect ecological, evolutionary, epidemiological, adaptive and geographical differences. Certain *Borrelia* species are more common to mammalian, avian or reptilian hosts [36,37], for example. Today, all recognized *B. burgdorferi* sl species can be divided into four ecological groups: a) species adapted to mammals (i.e. *B. afzelii, B. carolinensis*); b) species adapted to birds (i.e. *B. garinii, B. americana*); c) species adapted to reptilian hosts (i.e*. B. lusitaniae*) or d) species with no specialized hosts (i.e., *B. burgdorferi* ss). Such separation is not, however, absolute. Occasionally, different *Borrelia* species have been detected in ticks found on “inappropriate hosts”, whose complement normally eliminates mentioned species during the blood meal.

The key determinant of spirochete/host interaction has been associated with the complement regulator-acquiring surface proteins encoded by members of the *erp* gene family [36,38-40]. The general function of the *erp* gene family is to bind the host-derived complement control proteins in a species-specific pattern [41]. The *erp* genes represent prophage genomes [42] that are employed in reshuffling of genetic material among *Borrelia* strains, holding the key to the adaptive radiation of *Borrelia* species [43]. The dynamics of infection in host populations will determine the opportunity for mixing of different genotypes, allowing the horizontal gene transfer of genetic material, and triggering genetic changes in the *Borrelia* complex [43].

Kurtenbach and colleagues suggested that the diversity and evolution of LB spirochetes cannot be attributed to tick diversity, but appears to be driven mainly by the host [36]. The diversity of *Borrelia* species associated with rodents, which have migration rates of 200–300 meters per generation, is much lower than in species associated with birds [33]. This conclusion is also supported by the complex population structure of *Borrelia garinii* in subarctic Eurasia. *B. garinii* is thought to be genetically and antigenically the most heterogeneous species among *B. burgdorferi* sl complex because of its association with passerine and marine birds [15]. *Borrelia* has the recombination system needed for genetic exchange. Genetically diverse strains of *Borrelia* are often found within the same tick or same vertebrate host and this gives a wide opportunity for genetic exchange [44-46].

How does the genomic diversity within a *Borrelia* population originate and how is it maintained? Is the appearance of a new species determined by selective pressure from the vertebrate immune response, point mutations or horizontal gene transfer among sympatric genomes? Here we present the analysis of a small group of 8 single-bird-associated *Borrelia* strains primarily assigned to *B. americana*. Combined MLSA and MLST analyses revealed that level of divergence among 8 strains is higher than what would be expected for strains within a single species. We report the cross-species recombination that led to incorporation of the *B. burgdorferi* ss housekeeping gene *nifS* into the homologous locus in strain SCW-30 h. Our results support the hypothesis that recombination maintains a majority of sequence polymorphism within *B. burgdorferi* sl populations [47].

## Methods

### Ticks and *Borrelia*

Eight *Borrelia* strains were isolated from two developmental stages of the hard tick *Ixodes minor* collected from a single Carolina Wren (*Thryothorus ludovicianus*) captured at the Wedge Plantation, Charleston County, South Carolina, USA, in November of 1994. Strains SCW-30a, SCW-30b, SCW-30c and SCW-30d, were isolated each from an individual pool of 3 *I. minor* larvae. Strains SCW-30e, SCW-30f, SCW-30 g and SCW-30 h were isolated each from an individual *I. minor* nymph. Conditions for *Borrelia* cultivation were described elsewhere [30,31].

### Primers used in this study

Previously described PCR primer sets were used for amplification of 5S-23S IGS [48], 16S rRNA [49], *flagellin*[50], *p66*[50], *ospC*[51] and 16S-23S ITS [51]. The MLST scheme was used for amplification and analysis of 8 housekeeping genes: *clpA, clpX, nifS, pepX, pyrG, recG, rplB* and *uvrA*[52]. The amplification conditions for all selected genomic loci were strictly as described.

### General analysis of *Borrelia* isolates

DNA purification, PCR amplification, sequencing and sequence analysis were conducted according to our previously described protocol [31] and MLST scheme developed by Margos *et al.,* 2008 [52]. Total DNA from cultured spirochetes was purified using the DNeasy Blood and Tissue kit (Qiagen, USA). The MasterTaq Kit (Eppendorf, Germany) was used for amplification of selected loci. The purified PCR products were submitted for direct sequencing to the University of Washington High-Throughput Genomic Unit (Seattle, USA). Sequencing was conducted in both directions, using the same primers that were used for amplification of each locus. Sequences determined in this study have been deposited into GenBank.

The restriction fragment length polymorphism (RFLP) analysis of borrelia 5S-23S IGS and *flagellin* gene was done *in silico* using the free software available at http://insilico.ehu.es[53] as it was previously described [31]. The 5S-23S IGS was digested with *MseI* and *DraI* restriction endonucleases; *flagellin* gene was digested with *HapII, HhaI, HincII, CelII*, and *DdeI*. Obtained RFLP patterns were compared with patterns already published [13,47,54].

### Phylogenetic analysis

We sequenced 14 genomic loci for each *Borrelia* strain: i.e., 16S rRNA, a 5S-23S IGS, a 16S-23S ITR, *flagellin*, *p66*, *ospC* and housekeeping genes, *clpA, clpX, nifS, pepX, pyrG, recG, rplB,* and *uvrA*. Twelve loci were ultimately included in the phylogenetic analysis; *ospC* and 16S-23S ITS were excluded due to the high levels of polymorphism and recombination.

Sequences were aligned using Clustal X [55]. Data were evaluated for fit to 24 evolutionary models using MrModeltest [56]. The most-parameterized model that best fits the data at each locus was selected and evaluated by either the likelihood ratio test or Akaike Information Criterion [57]. Phylogenetic analyses were performed using Bayesian reconstruction methods, with the underlying model of evolution set to the chosen model in the program MrBayes 3.1. Selected models were: GTR + G for *clpA* (579 bp) and *nifS* (564 bp) loci, GTR + I + G for *clpX* (624 bp), *pepX* (570 bp), *pyrG* (603 bp), *recG* (651 bp), *rplB* (624 bp) and *uvrA* (570 bp), HKY + G for 5S-23S IGR (275 bp), HKY + I for *flagellin* gene (487 bp), and GTR + I for *p66* (315 bp) and 16S rRNA (1363 bp) [58,59]. The Markov Chain Monte Carlo (MCMC) analysis was run for 10 × 10^6^ generations, sampling trees every 1000 generations, using 4 Markov chains (default heating values). Stationarity of the MCMC was evaluated using the “Are We There Yet” (AWTY) software [60] that plots the cumulative posterior probabilities for each tree. Two to three thousand burn-in trees generated before the point, at which these values stabilized, were discarded. The fifty percent majority rule consensus tree for the estimated posterior distribution of trees (with burn-in trees truncated) was assembled for each locus, using MrBayes [59]. The consensus trees for each of twelve genes (excluding *ospC* and 16S-23S ITS) were not congruent, and thus an overall pattern of relatedness could not be inferred using these gene-trees alone.

The most common approach to inferring relationships across multiple genetic loci is to combine outcomes of individual gene trees into multi-locus analysis. The Bayesian estimation of concordance among gene trees (BUCKy) approach [61], which makes no assumptions about the source of reticulation in gene tree histories was used here. BUCKy uses, as input data, the complete tree files generated by the Bayesian analysis of each individual locus, in the format generated by MrBayes [59]. BUCKy generates a sample of gene trees from the joint distribution of gene trees, from which concordance factors (CFs) are estimated with credibility intervals. The CF ranges from 0.0 to 1.0. BUCKy implements a consensus method based on unrooted quartets and which consistently identifies the species tree [62]. We ran BUCKy at several levels of α to evaluate how much effect choice of this parameter value would have on the results. The final analysis selected for use was run with an α of 1, a reasonable intermediate between 0 and infinity [63], using 4 heated chains in the MCMC analysis.

### Nucleotide sequence accession numbers

Sequences determined in this study have been deposited into GenBank and given the indicated accession numbers (numbers for each isolate are given for sequenced genomic loci in the following order: 5S-23S intergenic spacer, 16S rRNA, *flagellin*, *p66*, 16S-23S ITS, and *ospC*): HM802215, HM802221, HM802227, HM802233, HQ012507 and HM852908 for SCW-30a; HM802216, HM802222, HM802228, HM802234, HQ012502 and HM852909 for SCW-30b; HM802217, HM802223, HM802229, HM802235, HQ012503 and HM852910 for SCW-30c; HM802218, HM802224, HM802230, HM802236, HQ012504 and HM852911 for SCW-30d; HM140981 and HM146415 for 16S-23S intergenic spacer and *ospC* gene of SCW-30e; HM802219, HM802225, HM802231, HM802237, HQ012505 and HM852912 for SCW-30f; HM140982 and HM146416 for 16S-23S intergenic spacer and *ospC* gene of SCW-30 g and HM802220, HM802226, HM802232, HM802238, HQ012506 and HM852913 for SCW-30 h. Accession numbers for 5S-23S IGS, 16S rRNA, *flagellin* and *p66* of isolates SCW-30e and SCW-30 g were presented earlier in Rudenko *et. al*, 2009 [32]. Sequences of housekeeping genes of all strains from SCW-30 group were submitted as 8 sets to the PopSet database and are accessible in GenBank using Entrez PopSet numbers: 358009204 for *clpA* genes, 358009220 for *clpX* genes, 358009236 for *nifS* genes, 358009252 for *pepX* genes, 358009268 for *pyrG* genes, 358009284 for *recG* genes, 358009300 for *rplB* genes and 358009316 for *uvrA* genes.

### Control strains of *Borrelia burgdorferi* sensu lato species used in analysis

The sequences of corresponding genomic loci of following control strains available in GenBank were used in the phylogenetic analyses: *B. burgdorferi* sensu stricto B31; *B. afzelii* VS461; *B. americana* SCW41; *B. andersonii* 21133; *B. bavariensis* PBi; *B. bissettii* DN127; *B. californiensis* CA443; *B. carolinensis* SCW22; *B. garinii* 20047; *B. japonica* HO14; *B. kutrenbachii* 25015; *B. lusitaniae* PotiB2; *B. sinica* CMN3; *B. spielmanii* A14S; *B. tanukii* HK501; *B. turdi* Ya501; *B. valaisiana* VS116; *B. yangtze* R5 and genomospecies 2 CA-2.

## Results

The eight strains isolated from *Ixodes minor* larvae and nymphs collected from a single Carolina Wren were analyzed previously at six genomic loci, 5S-23S IGS, 16S rRNA, *flagellin*, *p66*, 16S-23S ITS and *ospC*[30]. At all six loci 8 strains clustered as larvae-associated “b/c/d” group (SCW-30b, SCW-30c, and SCW-30d), nymph-associated “e/f/g” group (SCW-30e, SCW-30f, and SCW-30 g) and standalone nymph-originated SCW-30 h strain. In a previous study, strains SCW-30e and SCW-30 g were assigned to *B. americana*[32]. In the same study strain SCW-30f was also assigned to this species by preliminary analysis of the 16S rRNA gene [32]. The larvae-originated strain SCW-30a alternately clustered with the *B. americana* “e/f/g” and “b/c/d” clusters, depending on the locus analyzed.

PCR of the 5S-23S IGS locus resulted in amplicons of 3 different sizes across the 8 samples: 254 bp for SCW-30a, SCW-30e, SCW-30f and SCW-30 g, 253 bp for SCW-30 h and 225 bp for SCW-30b, SCW-30c and SCW-30d strains. *MseI* and *DraI* RFPL patterns of SCW-30e, SCW-30f, SCW-30 g and SCW-30 h corresponded to those described for *B. americana* subgroup A and B [32]. The RFLP pattern found at this locus in SCW-30b, SCW-30c and SCW-30d could not be associated with any known *Borrelia* species and represents four *MseI* restriction fragments, 107, 52, 38, and 28 bp, and three *DraI* restriction fragments, 144, 53, and 28 bp. The *DraI* RFLP pattern for SCW-30a was identical to that of *B. americana*, but the *MseI* pattern in that strain was different and contained 5 fragments of 107, 80, 38, 16, and 13 bp instead of the 6 specific to *B. americana*[32].

*In silico* RFLP analysis of the 488 bp *flagellin* gene involved sequence digestion with *HapII*, *HhaI*, *HincII*, *CelII*, and *DdeI* restriction endonucleases. Amplicons of SCW-30e, SCW-30f, SCW-30 g and SCW-30 h revealed the pattern specific for *B. americana*[32]. RFLP patterns in SCW-30a, SCW-30b, SCW-30c and SCW-30d at this locus were identical, but unknown, resulting in two fragments in case of restriction with *DdeI* only (338 and150 bp).

In the *p66* locus, SCW-30b, SCW-30c and SCW-30d strains showed 99.7-100% similarity among themselves only; SCW-30e, SCW-30f and SCW-30 g strains revealed 99.0-100% similarity to the *B. americana* type strain and to SCW-30 h; strain SCW-30a was 98.1-98.7% similar to both the “b/c/d” and the “e/f/g” groups of strains.

All 8 strains were 99.6-100% similar on the 1,316 bp amplicon of 16S rRNA.

Larvae-originated SCW-30b, SCW-30c and SCW-30d were 100% identical at 16S-23S ITS locus. Previously identified as *B. americana,* SCW-30e, SCW-30f, SCW-30 g, and SCW-30 h showed 99.88 -100% similarity among themselves. Strain SCW-30a showed the lowest similarity, 85.63-88.08%, to the rest of strains at this locus.

Strains SCW-30a, SCW-30b, SCW-30c and SCW-30d were identical at the *ospC* locus and showed no significant similarity to known *ospC* types. The *ospC* genes of SCW-30e, SCW-30f and SCW-30 g were 97-99% similar to those of other *B. americana* strains. SCW-30 h *ospC* was 100% identical to one of *B. americana* type strain SCW-41^T^ and revealed the high similarity to *ospC* allele B of *B. burgdorferi* ss strains that are widely distributed in southeastern United States [64].

Results of analysis of 6 genes using MLSA scheme from our previous studies [31,32] was insufficient to resolve the taxonomic status of the strains from SCW-30 group revealing apparent recombination of genetic material among fast evolving genomic loci.

Sequences of 8 housekeeping genes *clpA, clpX, nifS, pepX, pyrG, recG, rplB* and *uvrA* from 8 SCW-30 strains were compared with the allelic profiles using “virtual isolate collections centers” [35] from the online MLST database (http://www.mlst.net) [65]. The MLST database currently contains data for approximately 1,200 *Borrelia* strains comprising most of the described *B. burgdorferi* sl species from all over the world which have been resolved into >300 sequence types (ST’s) [50]. We found that only strains SCW-30f and SCW-30 g carried the same alleles as the type strain of *B. americana* SCW-41^T^. Strain SCW-30e was broadly variable at the housekeeping loci (Table 1). The similarity of strains SCW-30b, SCW-30c and SCW-30d were below the cut-off value for species assignment at 4 of the 8 loci analyzed*, pepX, pyrG, recG* and *uvrA* (Table 2). The divergence of strain SCW-30 h was the highest among the group. Cross-species recombination was detected with this method of comparison, and seemed to be the result of the incorporation of a *B. burgdorferi* ss housekeeping gene, *nifS*, into the homologous locus of strain SCW-30 h. This allele is specific to *B. burgdorferi* ss strains widely distributed in the United States and in Canada (borrelia.mlst.net).

**Table 1:** Genetic divergence among individual housekeeping genes of SCW-30 strains and estimated allelic profiles

	***clpA***	***clpX***	***nifS***	***pepX***	***pyrG***	***recG***	***rplB***	***uvrA***
SCW-41^Ta^	176^c^	141	127	154	159	159	136	150
SCW-33^b^	175	140	126	153	158	158	135	149
SCW-30a	175 (98.4%)	141 (99.7%)	127 (99.3%)	154 (98.6%)	159 (99.7%)	159 (99.1%)	136 (99.2%)	150 (99%)
SCW-30b	175 (98.4%)	141 (99%)	127 (99.3%)	**153 (97.5%)**	**159 (97.8%)**	**159 (98.2%)**	136 (99.5%)	**150 (98%)**
SCW-30c	175 (98.4%)	141 (99%)	127 (99.3%)	**153 (97.5%)**	**159 (97.8%)**	**159 (98.2%)**	136 (99.5%)	**150 (98%)**
SCW-30d	175 (98.4%)	141 (99%)	127 (99.3%)	**153 (97.5%)**	**159 (97.8%)**	**159 (98.2%)**	136 (99.5%)	**150 (98%)**
SCW-30e	175 (98.4%)	141 (99.8%)	127 (98.6%)	154	**159 (98.2%)**	159 (98.5%)	136 (99.8%)	**150 (98%)**
SCW-30f	176 (99.7%)	141	127	154	159	159	136	150
SCW-30 g	176 (99.7%)	141	127	154	159	159	136	150
SCW-30 h	176 (99.7%)	141 (98.4%)	3 (98.4%)	154 (98.9%)	**159 (97.5%)**	**159 (97.5%)**	135 (99.5%)	**150 (98%)**

The percentage of identity of housekeeping genes of SCW-30 strains to the closest alleles available in MLST database are presented in the Table 1.

SCW-41^Ta^ is a type strain of *Borrelia americana* and represents subgroup A (ATCC BAA-1877; DSM 22541).

SCW-33^b^ represents *B. americana* subgroup B.

176^c^ -unique allele numbers of housekeeping genes in MLST database. Strain allelic profile is a composition of 8 numbers presented in a single row. Marked in bold are values that are below or equal to a cut-off value of 0.170 determined for the MLST scheme based on eight chromosomally located housekeeping genes [26].

**Table 2:** Genetic divergences among SCW-30 strains based on 8 concatenated housekeeping genes

	**SCW-30a**	**SCW-30b**	**SCW-30c**	**SCW-30d**	**SCW-30e**	**SCW-30f**	**SCW-30 g**	**SCW-30 h**	**SCW-41**^**T**^
SCW-30a		98,9	98,9	98,9	98,5	99,1	99,1	**98**	99,1
SCW-30b	1,1		100	100	98,5	**98,3**	**98,3**	**97,7**	**98,3**
SCW-30c	1,1	0		100	98,5	**98,3**	**98,3**	**97,7**	**98,3**
SCW-30d	1,1	0	0		98,5	**98,3**	**98,3**	**97,7**	**98,3**
SCW-30e	1,5	1,5	1,5	1,5		98,9	98,9	**98,2**	98,9
SCW-30f	0,9	1,7	1,7	1,7	1,1		100	98,4	99,9
SCW-30 g	0,9	1,7	1,7	1,7	1,1	0		98,4	99,9
SCW-30 h	2	2,4	2,4	2,4	1,8	1,6	1,6		98,4
SCW-41^T^	0,9	1,7	1,7	1,7	1,7	0,1	0,1	1,7	

SCW-41^T^ is a type strain of *Borrelia americana* (ATCC BAA-1877; DSM 22541).

Marked in bold are values that are below or equal to a cut-off value of 0.170 determined for the MLST scheme based on eight chromosomally located housekeeping genes [26].

Eight concatenated housekeeping gene sequences from each SCW-30 strain (2,752 bp/strain) were aligned with 18 available in MLST database sequence types (ST) for type strain of known *Borrelia* species and genomospecies 2: ST1 (*B. burgdorferi* ss), ST64 (*B. lusitaniae*), ST70 (*B. afzelii*), ST83 (*B. garinii*), ST84 (*B. bavariensis*), ST95 (*B. valaisiana*), ST152 (*B. yangtze*), ST159 (*B. spielmanii*), ST272 (*B. bissettii*), ST280 (*B. kurtenbachii*), ST388 (*B. andersonii*), ST447 (*B. californiensis*), ST449 (*B. americana*), ST450 (*B. carolinensis*), ST453 (*B. japonica*), ST454 (*B. tanukii*), ST455 (*B. turdii*) (not shown). Genetic distance between SCW-30 group and all known species, except *B. americana*, was significant and far below the cut-off value of 0. 170 determined by MLST scheme [52]. Sequence pair distance analysis revealed that strains SCW-30b, SCW-30c and SCW-30d were identical among themselves. Strains SCW-30f and SCW-30 g were identical between themselves and definitely belong to *B. americana* (Table 2). The cut-off value 0.170 for species determination for this scheme was exactly the one that showed genetic distance between SCW-30b, SCW-30c and SCW-30d and *B. americana* strains. Based on analysis of concatenated sequences strain SCW-30 h represents a species distinct from that of SCW-30a, SCW-30b, SCW-30c and SCW-30d. Analysis based on individual housekeeping genes put SCW-30 h outside of *B. americana* group (Table 1).

Significant incongruence was observed in results of analysis of fast evolving and slow evolving genes or non-coding genomic loci of SCW-30a strain. This could reflect the recent recombination or re-assortment of sequence variations within SCW-30 group or another *Borrelia* population that we did not detect in this study. Analysis of concatenated housekeeping genes assigned SCW-30a to *B. americana* species, though as rather highly divergent member. This conclusion might not be definite yet.

### Phylogenetic analysis

**Figure 1:**
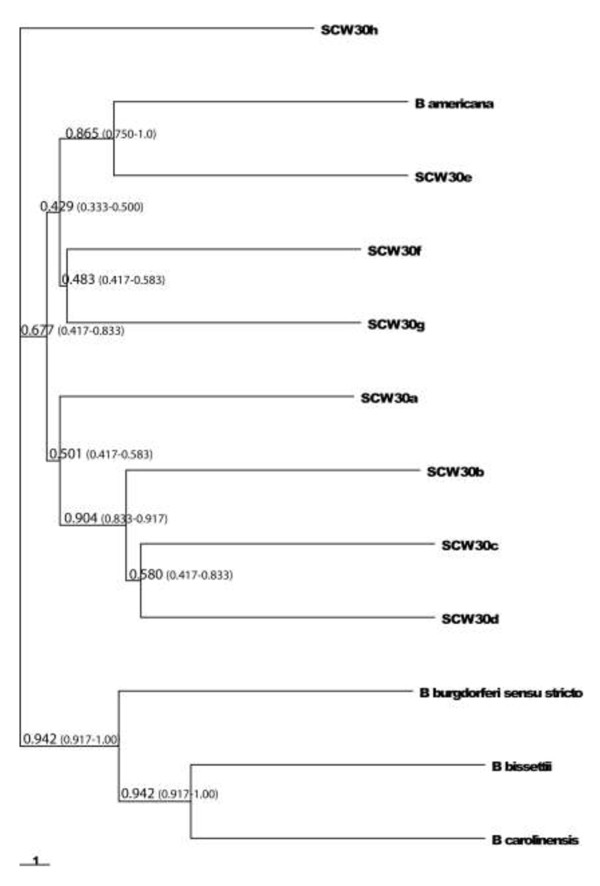
**Population tree of control *Borrelia *species and strains from SCW-30 group.** Fast and slow evolving plasmid and chromosome located genes and non-coding region were included into the analysis. Bayesian concordance analysis was conducted using BUCKy [63] with a value of 1.0 for α, the parameter that summarizes prior expectations of the amount of concordance among gene trees. Each split is annotated with the posterior mean sample-wide concordance factor and its 95% credibility interval. The concordance factor shows the proportion of the sample trees for which the split is true, ranging from 0.0-1.0. Branch lengths are in coalescence units.

The resulting population and consensus trees from the concordance analysis are shown in Figure 1. This is a combined analysis of fast and slow evolving, plasmid- and chromosomally-located genes and non-coding regions of 8 SCW-30 strains (excluding *ospC* and 16S-23S ITS). The reported sample-wide concordance factors on the tree (CFs) are not comparable to posterior probabilities or bootstrap support values, and thus they are not interpreted as a normal support statistic would be. They are used as information about the status of the clade in question. Values around 0.5 and above indicate that most of the trees in the sample set contain that clade, and that there is no predominant discordant clade configuration.

The type strain of *B. americana* clusters with SCW-30e forming a very well-supported clade, indicating that these are likely very similar or very recently diverged strains. SCW-30f and SCW-30 g cluster together with moderate support, and together with *B. americana* and SCW-30e form a moderately well-supported clade “e/g/f”. Strains SCW-30b, SCW-30c and SCW-30d form an extremely well supported clade, with a concordance factor above 90%. The arrangement of SCW-30c and SCW-30d within the clade is less well supported, but the three strains clearly share histories strongly. SCW-30a clusters with SCW-30b, SCW-30c and SCW-30d, but with only moderate support forming a clade “a/b/c/d”. SCW-30 h is located outside the “a/b/c/d” and “e/g/f” clades and its placement is moderately well supported (>67% but with a wide confidence interval).

## Discussion

Comparison of SCW-30 strains with the control samples makes it clear that either there is ongoing changes among these strains, or that the divergence is very recent. The concordance among gene trees for control samples as separate and distinct clades is extremely high. However, concordance among gene trees for most of the clusters of SCW-30 strains is less clear. The clades that stand out as distinct are *B. americana* + SCW-30e, than SCW-30b, SCW-30c, SCW-30d and, SCW-30 h. Branch lengths make it clear that genetic divergence of these strains is as great as divergence among other clearly designated distinct species. Traditional rules of *Borrelia* taxonomy would support the claim that some of SCW-30 strains could have the status of new species, distinct from *B. americana*. However, the ongoing changes (from whatever source) and incongruence makes it difficult to determine if new species status should be assigned to selected SCW-30 strains, even after extended molecular analysis of 14 genomic loci of different functionality, which is the biggest known attempt other than the whole genome sequencing. The support for any cluster at this point would be ambiguous.

Local *Borrelia* populations show established biogeographic structure, often including a significant number of distinct genomic groups and are highly diverse, often co-infect a single tick species or vertebrate host, and co-exist in sympatric fashion [44,51,66-68]. MLST and whole genome sequencing of different *Borrelia* species has confirmed that horizontal exchange of genetic information is pervasive across the *Borrelia* genome, occurs frequently between different genospecies and is more frequent than point mutations [47]. The origin of the high diversity within a local *Borrelia* population is an open subject for discussion. Several hypotheses have been proposed and one suggests that the diversity and evolution of LB spirochetes appears to be driven mainly by the host [36], and distinct clonal groups are maintained by host specialization [44,69].

The importance of birds as reservoir hosts in the ecology of LB around the world is clearly recognized today. Birds are capable of transmitting the highest number of known genotypes, albeit at different frequencies [70]. Multiple studies have confirmed the involvement of different bird species in the enzootic maintenance of *B. burgdorferi* sl species in endemic areas [1,10,17,40,54,71-78]. Migratory passerine birds have been shown to be responsible for spreading *Borrelia* infected ticks within and between continents, establishing new foci for Lyme borreliosis [13,16,66,77-79].

Passerines are parasitized by both tick larvae and nymphs [80]. Typically, in the northern populations of *I. scapularis* and *I. ricinus,* infected nymphs transmit spirochetes to hosts that subsequently infect larval ticks. Since the discovery of Lyme borreliosis, the evidences of transovarial transmission of *B. burgdorferi* sl were presented and it was believed until recently that larval ticks may, infrequently, obtain the LB spirochete by transovarial transmission, with the prevalence of less that 1% [81]. A recent literature review and observations have indicated that the transovarial transmission of the LB spirochete does not exist and was confused for years with the transovarial transmission of the antigenically and phylogenetically related *Borrelia miyamotoi*[82]. This fact leaves the only possibility for larvae to get infected with LB spirochete - the host. The relatively high prevalence of *Borrelia*-infected larvae collected from birds (from 3–3.1% [82,83] to 29% [14,83]) indicates that they became infected while feeding on birds. When the spirochetes persist in a bird for a long time, birds become an amplifier of *B. burgdorferi* sl, transmitting the pathogen to a greater number of ticks. Larvae maintain infection through the molt, giving rise to a new population of infected host-seeking nymphs. The efficiency of this cycle leads to a high prevalence of *B. burgdorferi* sl infection in questing nymphs, and a high public health risk in the region [84].

The transmission of *B. burgdorferi* sl between ticks and vertebrate hosts is a complex process. The interaction of *Borrelia* with the alternative pathway of the host’s complement system is considered to be the key determinant of spirochete-host association [36,85-88]. As part of the innate immune system, the alternative pathway can rapidly respond to pathogens before antibodies are generated [36]. A clear pattern of resistance or sensitivity of spirochetes to host complement is correlated with patterns of transmissibility. Spirochetes that are sensitive to the complement of a particular species are lysed by the host complement in the gut of the feeding tick before they are transmitted to the hosts. Selective survival of *B. burgdorferi* sl in the tick midgut, depending on the source of serum and the genetic background of the bacteria, can manifest itself in the selective replacement of *Borrelia* strains during the tick life cycle [36]. The high diversity of local spirochete populations has often been connected to host specialization or to the coexistence of multiple genospecies in the region with pervasive recombination among sympatric genomes [11,47]. Multiple-niche polymorphism, a form of balancing selection, can maintain diversity within the population [44].

Analysis of the 8 *Borrelia* strains cultured from *I. minor* larvae and nymphs and reported here showed high heterogeneity among the isolates using different methods of analysis. Previous MLSA of 5 genomic loci of *B. americana* strains separated closely related members into two subgroups, A and B [32]. In this study, combined analysis that involved several methods and 14 genomic loci of SCW-30 strains showed a significant divergence among the strains isolated from 2 developmental stages of *I. minor*, feeding on a single bird. Earlier studies show that if more than one infected tick was collected from a single bird host, all ticks harbour the same spirochete species [83]. Three strains, SCW-30e, SCW-30f and SCW-30 g, cultured from *I. minor* nymphs support this observation, representing diverged strains of the same species. However, analysis of four *Borrelia* strains isolated from *I. minor* larvae indicated that they were distinct from *B. americana* and other known spirochete species at majority of genomic loci analyzed. While strains SCW-30b, SCW-30c and SCW-30d clustered together, localization of SCW-30a was incongruent over the whole spectrum of analyzed loci, indicating a probable high level of horizontal genetic exchange among all 8 strains connected to a single bird reservoir host. Even though the recombination does not often occur in a core region of the *Borrelia* genome [89], a single event of gene conversion was registered in strain SCW-30 h. A *nifS* gene from *B. burgdorferi* ss, a species that is widely distributed in the United States and Canada, was incorporated into the homologous locus of strain SCW-30 h. This might be possible only in the presence of sympatric genome, either by transmission through a bird host that tick nymphs fed on, or by transmission through the molt stage of larvae that fed previously on a host infected with *B. burgdorferi* ss.

A laboratory study of *I. minor* indicated that both nymphs and larvae feed for 4 days on average when fed on laboratory white mice (*Mus musculus*). Adults were reluctant to feed on the mice, but readily fed on eastern woodrats (*Neotoma floridana*) [90]. In nature *I. minor* feeds on a variety of mammals and birds including the cotton mouse, house mouse, cotton rat, cottontail rabbit, eastern rice rat, eastern grey squirrel, eastern spotted skunk, eastern woodrat, and the bird species, Carolina wren, house wren, and the eastern towhee see ref. [90], establishing the possibility of harvesting and amplification of multiple spirochete species.

We have cultured a large number of *Borrelia* strains from 8 bird species: Carolina wren (*Thryothorus ludovicianus*), downy woodpecker (*Picoides pubescens),* white-eyed vireo (*Vireo griseus),* Swainson’s thrush (*Catharus ustulatus*), American redstart (*Setophaga ruticilla*), northern water thrush (*Parkesia noveboracensis*), pine warbler (*Setophaga pinus),* and northern cardinal (*Cardinalis cardinalis*) [91]. Four *I. minor* nymphs, collected from a Carolina Wren, had a chance to pick up *B. americana* from various hosts during the larval feeding or during their current nymph feeding. *I. minor* larvae collected from the same bird were feeding for the first time, picking up *Borrelia* species from the same bird host. It is not unusual that the vector, the reservoir host or LB patient maintain multiple spirochete species [92-99]. However, it is difficult to explain the unprecedented level of diversity among single-bird host-associated spirochete strains, specifically those isolated from larvae, considering the recent claim that transovarial transmission of *B. burgdorferi* sl does not exist [82].

Our previous studies of LB spirochetes showed very small if any diversity of strains isolated from different vector ticks or rodent hosts, whether it was a group of *B. burgdorferi* ss strains or distinct spirochete species [31,64,91]. *B. burgdorferi* sl species are adapted to hosts and this adaptation is driven by host complement. The dynamics of infection of the host supports the mixing of different genotypes and the horizontal exchange of genetic information. The striking divergence of *Borrelia* strains associated with a single bird reservoir that we present here supports the earlier hypothesis that vertebrate hosts are the key determinants in the diversity of Lyme disease spirochete [36].

## Conclusions

Genetically diverse *Borrelia* strains are often found within the same tick or same vertebrate host and this gives a wide opportunity for genetic exchange**.** We report a case of cross-species recombination that led to the incorporation of an allele of a housekeeping gene from a *B. burgdorferi* sensu stricto strain, the primary causative agent of LB, into the homologous locus of bird-associated strain. Our results add to the recent hypothesis that recombination maintains a majority of sequence polymorphism within *Borrelia* populations due to re-assortment of pre-existing sequence variants. Even though our findings of increased diversity among 8 strains cultured from ticks that fed on a single bird could be the exception rather than the rule they support the theory that diversity and evolution of LB spirochetes is driven mainly by the host.

## Competing interests

The authors declare that they have no competing interests.

## Authors’ contributions

NR and MG designed the experimental scheme and carried complete laboratory work: i.e., strains cultivation and maintenance, DNA purification, PCR amplification and analysis, sequence analysis, RFLP analysis, sequence alignments, results evaluation and drafted the manuscript. NMB conducted phylogenetic analysis, evaluated results, drafted the figures and edited the manuscript. LG designed the study, evaluated results and edited the manuscript. JHO isolated bird-associated samples, cultured SCW-30 strains, evaluated results and drafted the manuscript. All authors read and approved the final version of the manuscript.
